# Tally Twenty-eight

**Published:** 1913-06

**Authors:** 


### EDITORIAL DEPARTMENT.

DR. F. E. DANIEL, Editor DR. R. H. L. BIBB, Associate Editor

DEPARTMENTS { EUGENICS: Drs. M. Duggan and T. Y. Hull.

WOMAN'S DEPARTMENT: Mrs. F. E. Daniel.

TALLY TWENTY-EIGHT.—This issue of the Journal completes its twenty-eighth year under one, the original, management. It has cause to congratulate itself on its success, and to thank its patrons for their steadfast friendship and loyal support. The editor has grown gray, but he abates not a jot in his zeal in the cause of legitimate medicine, and the welfare and best interests of the regular profession. Whether we have done any good or not is for others to say and remains to be seen. We have tried. We have ever been conscientious in the endeavor to inculcate a high idea of professional character, a pride in the achievements of those who have gone before, a reverence for those who have made medicine illustrious, an appreciation of the honor and dignity of the profession. We begin Volume 29 (July) with undiminished interest and zeal in the cause and a determination to keep the Red-Back in the forefront, on the firing line, as full of zeal, fun and fight as when these frosty locks were like the raven, John, and this bonny brow was "brent," whatever that may be.

And speaking of professional character, the thought comes to me that the subject should be a part of every lecture course. The standard should be fixed high and the young man inspired to attain it. Hippocrates set the example, and if one lives up to the oath which he exacted of his followers they will not disgrace their calling. The reflection comes, too, that since the "Red Back" was founded twenty-eight years ago and made the exemplar of professional ethics for the Texas doctors, hundreds, yea thousands, of young men have been licensed to practice in Texas; yea, hundreds of the young men now practicing medicine in Texas were *born into the world* in that time. They have not been taught, mayhaps, and hence we hear complaint of "unprofessional conduct" now and then. I dare say thousands of them never read the oath, hundreds never heard of it. For their benefit I publish it herewith, and feel that I am doing a benefaction.

### The Hippocratic Oath.

I do solemnly swear by whatever I hold most sacred:

That I will be loyal to the profession of medicine and just and generous to its members;

That I will lead my life and practice my art in uprightness and honor;

That into whatsoever house I shall enter, it shall be for the good of the sick to the utmost of my power, I holding myself aloof from wrong, from corruption, from the tempting of others to vice;

That I will exercise my art solely for the cure of my patients and will give no drug, perform no operation for a criminal purpose even if solicited, far less suggest it;

That whatever I shall see or hear of the lives of men which is not fitting to be spoken, I will keep inviolably secret.

---

We reproduce in this issue a most remarkable paper which appeared in the *Charlotte (N. C.) Medical Journal* last month, and append without apology the following from the *Brownwood Bulletin*. It will be noted that Dr. Warren thinks that the use of tuberculin should be prohibited by law.

San Angelo, May 28.

Announcement was made Tuesday evening by Stanley Servier Warren, M. D., a member of the United States Reserve Corps and an ethical physician, that he was positive that phenol-perolatum, known commercially as corbolated vaseline, was an active therapeutic agent in the treatment of pulmonary tuberculosis and its use would aid in nature effecting a positive cure in the most advanced stages of the disease. Dr. Warren has treated many here during the past several years, using phenol-petrolatum or common vaseline both hypodermically and as an inunction. His former patients here will testify to its remedial aid in assisting nature in absolutely effecting a cure of the disease.

Dr. Warren stated this morning that he wished the world to know of his discovery and he also wished all to know that he has absolutely nothing to sell. He attributes his discovery to a visit at the hospital of the Southern Pacific railroad in Lower California a few years ago of an Englishman who was a beach-comber suffering with the white plague. The man came asking that something be given him to keep mosquitoes from eating what remained of his emaciated body. He was told to use carbolated vaseline. The man applied it all over his body and did so each night for several months.

He improved so rapidly in health that Dr. Warren began to make other tests, using vaseline. The results achieved were wonderful and he began to figure out in the hospital laboratory how to use phenol-petroleum hypodermically. This he eventually discovered Since coming to San Angelo he has used it both hypodermically and as an inunction with wonderful results.

Dr. Warren has seen army service in Cuba and governmental service in Panama. He is a graduate of the University of Pennsylvania, class of 1894. He has served interneships in two hospitals of Philadelphia. He is licensed to practice in the District of Columbia, Tennessee and Texas, as well as the Republic of Mexico, where he spent several years as surgeon for a Pittsburg syndicate in the Sierra Madre mountains in the State of Chihuahua. He was also stationed for a time at Fort Clark, near Brackettville, Texas. He is not only a member of the United States Medical Reserve Corps, but is an associate member of the Benjamin Rush Medical Society of Philadelphia and a member of the H. C. Wood Medical College Society of Philadelphia.

Dr. Warren resides north of San Angelo a few miles. He delights in spending his spare time in gardening and raising shrubbery. Tuesday evening he was honored by being elected an honorary member of the San Angelo Chamber of Commerce.

